# Time of Grain Supplementation and Social Dominance Modify Feeding Behavior of Heifers in Rotational Grazing Systems

**DOI:** 10.3389/fvets.2020.00061

**Published:** 2020-03-06

**Authors:** Gabriela Schenato Bica, Luiz Carlos Pinheiro Machado Filho, Dayane Lemos Teixeira, Karolini Tenffen de Sousa, Maria José Hötzel

**Affiliations:** Laboratório de Etologia Aplicada, Departamento de Zootecnia e Desenvolvimento Rural, Universidade Federal de Santa Catarina, Florianópolis, Brazil

**Keywords:** social hierarchy, cattle, resources, agonistic interactions, subordinate

## Abstract

Social hierarchy affects the access of animals to feed resources. On daily rotational pasture systems, supplementation time may influence feeding behavior. This trial was designed to test the effect of grain delivery time on the feeding behavior of heifers. Heifers divided into two groups according to breed (*n* = 15 Braford and *n* = 19 Jersey) were tested in a crossover design with two treatments: INITIAL—supplement at 8 am (entry time to a fresh paddock), and MIDDLE—supplement at 4 pm (middle time of paddock use). Animals entered a new paddock every morning, and grain supplement at 2 kg/animal/day was offered at the fence line (1 m/animal). Then, ingestive and other behaviors were registered by direct visual observation through scan sampling at 2-min intervals for 1 h after grain supply. Agonistic interactions were recorded continuously (instigator–victim) to build a social matrix whereby each heifer was defined as dominant, intermediate, or subordinate. Weekly pasture samples were collected according to the order that animals left the feeding area, using the hand-plucking technique, to determine crude protein and fiber content. Heifers spent more time grazing on the INITIAL treatment (*p* < 0.0001) but exhibited more behaviors on the MIDDLE treatment (*p* < 0.0001). Dominant heifers spent more time eating grain (*p* = 0.0008), whereas subordinate heifers spent more time grazing along the paddock (*p* = 0.0067), but not along the fence (*p* = 0.0008). The crude protein content of pasture samples was higher for the INITIAL treatment (*p* < 0.0001). Behavioral interaction occurred with respect to the order of leaving the feeding area, social rank, and crude protein consumed (*p* = 0.04). Subordinate heifers consistently grazed more and ate less grain supplement than dominant and intermediate heifers. However, when grain supplement was offered at the time animals entered the paddock, more grazing activity took place during supplement feeding, and subordinate heifers could select a high-protein diet. In the INITIAL treatment, this means that subordinate animals could benefit from the better pasture available, keeping a distance from dominant heifers, reducing agonistic interactions and likely improving their welfare.

## Introduction

Rotational grazing systems, as proposed by Andre Voisin, allow better use of the pasture, ensuring sufficient interval between two successive shearings for vigorous regrowth and ensuring that animals will forage at the optimal level (1). Despite the benefits of rotational grazing, farmers may need to offer feed supplements to the animals as a nutritional increment in times of pasture scarcity or as part of the diet of highly productive animals.

Supplementation at specific times of the day may supply the ruminant animal with an appropriate boost in energy and protein substrates, but may also alter grazing patterns (2, 3). Despite widespread use of dietary supplements, technical recommendations to farmers target a regular daily supply of feed to maximize weight gain or increase milk production. However, these recommendations mainly consider such aspects as animal category, nutritional requirements, stage of pastures, and cost of supplements, without taking into consideration the social behavior of the animals and the consequences of such behavior with respect to resource access. Cattle are social animals and organize themselves into hierarchies according to their willingness and ability to fight for resources (4). Social hierarchy affects individual access to resources, and dominant animals are known to exert pre-eminence over resources (4–6), especially when resources are limited (7). Social hierarchy thus affects drinking (8) and feeding behavior (9, 10).

Grazing behavior may also be related to diurnal changes in food quality (11). The circadian rhythm of forage increases soluble sugar concentrations during the day, which may explain why herbivores show a strong preference for afternoon, rather than morning, harvested forage (12). When instantaneous stocking rate is increased, more competition arises for food, and the forage availability per animal and animals' selectivity are reduced. In rotationally grazed paddocks, sward structure changes continually as grazing proceeds along the day, and as a result, changes in quantity and quality associated with the depletion of the sward have a detrimental effect on the bite mass and the intake rate (13). In this scenario, subordinate animals may have their access to feed limited, compromising their welfare.

Therefore, if dominant animals have priority over the use of resources, we raised to question how subordinate animals would behave under such conditions. We further asked what strategies might be used by both animals and farmers to mitigate the negative effects of social dominance to subordinate animals. In a rotational grazing system, we know that animals enter a new paddock every morning. Therefore, based on the animals' physiology, it would be logical to offer feed supplement in the late afternoon when pasture availability is decreased, and the animals are more motivated to obtain feed. However, we hypothesized that subordinate animals could graze the best patches, while dominant animals would eat feed supplement as long as it is offered when animals enter the new paddock. Thus, this study was designed to compare the effect of different delivery times (morning × afternoon) of grain supplement on the feeding behavior of heifers managed in a rotational grazing system.

## Materials and Methods

The study was conducted between June and August of 2016 (winter) at the Voisin's Rational Grazing (VRG) Unit of the Federal University of Santa Catarina Experimental Farm of Ressacada, Florianópolis, Brazil (17°40'25” S; 48°32'30” W). The VRG unit is a 24-ha pasture divided into 86 paddocks averaging 2,500 m^2^ and mainly composed of plants of the genus *Axonopus, Paspalum, Brachiaria, Pennisetum, Melinus, Setaria, Cynodon, Panicum, Hemarthria, Desmodium, Trifolium, Lotus, Arachis, Stylosanthes*, and *Lolium*. The study was performed in accordance with the Ethics Committee on Animal Use of the Federal University of Santa Catarina (CEUA/UFSC) under the approved protocol number 1004100516.

### Animals, Treatments, and Experimental Design

Before the study, the animals were routinely managed in two groups, according to breed: Braford and Jersey heifers without any feed supplementation. These breeds make up the herd of the experimental farm and are very representative of the herds in southern Brazil, where the Jersey breed is the most common breed for grazing milk production and the Braford breed is well-adapted to the region, being composed of zebu (3/8) and taurine (5/8) blood.

For the experiment, the separation between breeds was kept, and two groups were formed: 15 Braford heifers (group 1, averaging 316 ± 44 kg) and 19 Jersey heifers (group 2, averaging 232 ± 33 kg). Each group was first allocated to one of the treatments: INITIAL—supplement was offered at occupation time (8 a.m.); and MIDDLE—supplement was offered at middle occupation time (4 p.m.). The experimental design was a crossover. In each period, the animals had 5 days for habituation to observers and the experimental routine, followed by 35 days (each day in a new paddock) for data collection.

Animals were moved to a new paddock every morning with mineral salt and water *ad libitum*. Space availability per animal in the paddocks was ~145 m^2^/animal. Animals were identified by ear tags and individually marked with numbers on their bodies with black and green livestock markers (Raidex., Dettingen; Erms Germany). The supplement was a commercial ration for cattle (12% CP) and was offered on a daily basis of 2 kg/animal/day on the ground at the fence line in the morning or afternoon, according to treatment.

### Measurements

Data collection included observations of agonistic interactions and ingestive behavior, recorded simultaneously and in the two groups, as to avoid any environmental influence in their behavior. The agonistic interactions were continuously recorded, and the ingestive behavior was recorded by instantaneous scan sampling with a 2-min interval (14) twice a week for one uninterrupted hour from the moment the grain supplement was offered, resulting in 20 non-consecutive days of direct visual observation. Six trained observers switched groups within and between periods so that every person could observe the same number of times, groups, treatments, and periods, completely balancing the observations. The ethogram of the behaviors observed during the study is described in Table 1.

**Table 1:** Description of behaviors observed during the study.

**Behavior**	**Description**
Grazing along the paddock	Animal grazing along the paddock, with head down and the mouth below or at the level of the forage making movements of forage apprehension or grabbing forage; stationary or moving forward to new grazing patches
Grazing near the fence	Animal grazing as described above but along the fence line where the grain supplement was offered (feeding area)
Eating supplement	Animal eating grain supplement, with head down on the fence line and mouth on the supplement or above it while chewing
Other	When the animal performed an activity, either standing or lying, with the exception of the behaviors described above

*The ethogram was based on the definitions by Coimbra et al. (8)*.

All agonistic interactions during the 1-h observation period were recorded—displacements, threats, and other behaviors associated with a conflict or fighting between two individuals that involved an instigator and a victim, including, or not, physical contacts, resulting in the physical displacement of an animal (15). Then, a dominance index was calculated according to Kondo and Hurnik (16). An “S” value was calculated for each heifer relative to the others. Therefore, if animal “I” won over animal “J” in Xij interactions, and animal “J” won over animal “I” in Xji interactions, then Sij would correspond to Sij = Xij-Xji/|Xij-Xji|, always resulting in a value of −1, 0, or +1. Then the dominance index for heifer “I” (Si) would be the sum of S that animal had in each dyad. The dominance value for each individual was calculated as a result of the sum of all relationships of each animal with all other animals within the group. When two or more animals had the same “S” value (for example, cow 17 = cow 36), the tiebreaker was the result of direct confrontation between both animals.

A dominance index was constructed for each group based on the difference between the maximum and minimum dominance value, and then it was divided into three social categories: dominants (D) in the upper stratum, intermediates (I) in the middle, and subordinates (S) in the lower stratum of the index. Social hierarchy of each heifer and its dominance score are shown in Table 2.

**Table 2:** Dominance score (Score) and respective social hierarchy (SH) of each individual animal within each group.

**Group 1**			**Group 2**		
**Score**	**SH**	**Animal**	**Score**	**SH**	**Animal**
13	D	8	16	D	19
10	D	4	14	D	33
8	D	13	14	D	34
6	D	9	10	D	35
5	D	1	8	D	16
4	I	2	6	D	28
0	I	5	4	D	31
0	I	10	4	I	27
−1	I	12	2	I	29
−2	I	14	0	I	25
−4	I	6	−2	I	17
−6	S	7	−3	I	23
−9	S	15	−4	I	30
−12	S	3	−6	I	32
−12	S	11	−10	S	37
−			−11	S	20
−			−12	S	26
−			−12	S	36
−			−18	S	24

*SH: D for dominant, I for intermediate, and S for subordinate animal*.

To estimate the quality of the consumed diet (crude protein/CP and neutral detergent fiber content/NDF), weekly samples of pasture were collected accordingly by hand-plucking. Grazing simulation can be defined as harvesting a forage sample in the areas where the animals were grazing and simulating the morphological composition of the forage consumed by the heifers (17). In each group, six focal animals were selected for pasture collection. The first three animals (FIRST3) starting to graze and the last three animals (LAST3) leaving the feeding area were chosen. Samples were taken along the paddock immediately after grazing started (SAMPLE1) and then 1 h later (SAMPLE2). Each sample was conditioned in a tagged plastic bag, taken to the laboratory and dried in a forced-air buffer for 72 h at 55°C until constant weight. Then, samples were ground to pass a 1-mm screen in a Wiley mill before analysis using near infrared spectroscopy (NIR/MPA, “Multi-Purpose Analyzer,” Bruker Optics GmbH, Ettlingen, Germany). The FIRST3 and LAST3 data were also used for the analysis of the correlation between the order to leave the feeding area and start grazing and the social hierarchy of each individual.

### Statistical Analysis

Descriptive statistics were calculated using Microsoft® Excel® for Windows, and all other statistical analyses were conducted using SAS 9.3. The percentage frequency of behaviors was summarized over the days per period yielding one value for each animal per period. The Shapiro test was used on the model residual information, as well as the examination of the normal plot to evaluate the dataset for the normal distribution.

The effect of treatment and social rank on the percentage frequency of eating grain supplement, grazing on paddock, and grazing near fence line and along the paddock was analyzed using mixed procedures (Proc Mixed of SAS). The effect of treatment and social rank on the frequency of other behaviors was analyzed using generalized linear mixed models (Proc Glimmix of SAS). Treatment and social rank were included in the model as fixed effect, period as random effect, and gamma as the type of distribution. The effect of breed and interactions between treatment and social rank were removed from all models as they were not significant (*p* > 0.05). Results of eating grain supplement, grazing along the paddock, and grazing near fence line are reported as the least square means ± standard error (S.E.) of the percentage frequency; results of other behaviors are reported as least square means (95% confidence interval).

The relation between treatment order (FIRST3; LAST3), forage sample (SAMPLE1; SAMPLE2), social rank (dominant, intermediate, subordinate), and pasture contents as crude protein, acid detergent fiber, and neutral detergent fiber were analyzed using mixed procedure (Proc Mixed of SAS). Treatment, sample, order, and social rank were included in the model as fixed effects and period as random effect. Interactions were included in the models when they were significant (*p* < 0.05). Results are reported as least square means ± S.E.

The number of agonistic interactions (either instigator or victim) for each animal was summed per period (morning, afternoon). The effect of period and social rank on agonistic interactions was measured through analysis of variance (Proc GLM). Period and social rank were included as fixed effect and animal as the experimental unit. Interactions between period and social rank were tested and excluded from the model, as they were not significant (*p* > 0.05). Data are expressed as least square means ± S.E. of the number of agonistic interactions/animal.

## Results

Treatment did not affect the time heifers spent eating grain supplement or the time they spent grazing near the fence (feeding area), but it did affect the total time dedicated to grazing along the paddock (*p* < 0.0001) and other behaviors (*p* < 0.0001), as shown in Table 3. Heifers spent more time grazing on the INITIAL treatment and performed other behaviors more frequently in the MIDDLE treatment.

**Table 3:** Effect of treatment (INITIAL; MIDDLE) and social rank (dominant, intermediate, or subordinate) on behavior: eating grain supplement, grazing, and other behaviors [normal data: least square mean ± standard error; non-normal data: least square mean (95% confidence interval)].

**Behavior (%)**	**Treatment**			**Social rank**			
	**Initial**	**Middle**	***p*-value**	**Dominant**	**Intermediate**	**Subordinate**	***p*-value**
Eating grain supplement	28.6 ± 0.96	30.8 ± 0.96	0.1113	32.9 ± 1.12^a^	30.2 ± 1.12^a^	26.0 ± 1.32^b^	0.0008
Grazing on paddock	47. ± 7.15^a^	26.1 ± 7.15^b^	<0.0001	30.8 ± 7.27^a^	33.6 ± 7.27^a^	45.2 ± 7.45^b^	0.0020
Grazing near fence line	16.1 ± 1.94	19.0 ± 1.94	0.2867	23.8 ± 2.26^a^	18.9 ± 2.26^a^	9.9 ± 2.66^b^	0.0008
Other	2.1 (1.243–2.984)^a^	2.9 (2.071–3.812)^b^	<0.0001	2.4 (1.476–3.230)	2.6 (1.704–3.457)	2.6 (1.764–3.535)	0.1319

*Means with different letters in a row indicate significant differences (p < 0.05)*.

Regardless of treatment, social hierarchy influenced the feeding behavior of the group. Dominant animals spent more time eating grain supplement compared to subordinate animals (*p* = 0.0008), which, in turn, spent more time grazing along the paddock (*p* = 0.0067), but not along the fence (*p* = 0.0008).

The order to leave the feeding area and start grazing was inversely related to social status. Of the first three heifers leaving the supplement site, most (53.3%) were subordinate, whereas of the last three heifers leaving the supplement site, most (48.3%) were dominants.

A significant effect of treatment was observed on the crude protein content (INITIAL: 11.27 ± 1.5 vs. MIDDLE: 8.27 ± 1.47; *p* < 0.0001) of the forage collected as grazing simulation. An interaction was also noted among the order to leave the feeding area, social rank, and crude protein content (*p* = 0.04) (Table 4). The NDF content was higher for LAST3 compared to FIRST3 (FIRST3: 70.26 % ± 1.52; *p* > 0.05; LAST3: 73.74 % ± 1.53; *p* = 0.0367). Treatment, sample, order, and social rank were not related to FDA (39.28% ± 0.96; *p* > 0.05).

**Table 4:** Crude protein (%CP) content of hand-plucked pasture samples, according to the order of leaving the feeding area to start grazing (FIRST3; LAST3) and the social rank (dominant, intermediate, subordinate).

**Content/order**	**FIRST3**			**LAST3**		
	**Dominant**	**Intermediate**	**Subordinate**	**Dominant**	**Intermediate**	**Subordinate**
Crude protein (%CP)	12.3 ± 1.88^a^	8.5 ± 1.55^b^	10.2 ± 1.5^a^	9.2 ± 1.65	9.3 ± 1.54	8.9 ± 1.78

*Means with different letters in a row indicate significant differences (p < 0.05)*.

Treatment and social rank affected the number of agonistic interactions. Heifers performed fewer agonistic interactions on INITIAL (182.1 ± 14.03) compared to MIDDLE (249.2 ± 14.03; *p* < 0.01). Dominant heifers (247.7 ± 16.26) performed more agonistic interactions than subordinate heifers (182.72 ± 19.15; *p* < 0.05). Intermediate heifers (216.6 ± 16.26) performed a number of agonistic interactions similar to that of the other two (*p* = 0.2).

## Discussion

Treatment and social status affected grazing along the paddock. Heifers spent more time grazing along the paddock when grain supplement was delivered at the time of paddock entry (INITIAL), and subordinate heifers grazed longer than dominant and intermediate heifers during grain supplement feeding. On the other hand, no difference was noted between eating grain supplement and grazing near the fence line (where supplement was placed) relative to treatment. However, dominant and intermediate heifers ate more grain supplement and grazed longer near the fence line when compared to subordinate heifers, regardless of treatment.

As seen in a number of works, supplemental feeding may affect the total grazing time (18), and the time of supplementation is likely to affect grazing. For example, beef cattle grazed for a longer period when corn supplement was offered in the afternoon (19). Steers receiving supplement had the highest forage dry matter intake when supplement was offered at noon compared to 7 am and 4 pm (2). On the other hand, Sheahan et al. (20) concluded that supplementing cows in the morning or in the afternoon does not affect the time spent in grazing or dry matter intake.

All these studies were conducted in extensive grazing systems, considered whole herd behavior, and were focused on the total grazing time. Our study was focused on the effect of social status on grain supplement access and grazing time during supplement feeding. Moreover, heifers were in a rotational grazing system, entering into a new paddock every morning with fresh pasture available. The major grazing events occur in the early morning and late afternoon/early evening; the later grazing event is the longest and most significant in terms of herbage intake (21); according to this author, the dusk grazing event seems to be an adaptive feeding strategy to maximize daily energy acquisition, providing a steady release of nutrients throughout the night. Grazing behavior and intake are a multifactorial phenomenon and interact strongly with the morphological characteristics of grazed plants and the environment such as climate, the feed supply–demand balance, pasture composition, and grazing method, and the challenge is to present feed to grazing animals in ways that allow them to meet their dietary preferences, while also allowing high rates of animal production per hectare (22). Grazing time is affected by the grazing system, with lower grazing times on rotational systems compared to continuous systems, which may be attributed to the ability of cows to anticipate the timing of the daily movement of the electric fence and, correspondingly, reduce the time spent grazing residual herbage (23).

In our study, dominant heifers spent more time eating grain supplement and grazing along the fence line than subordinate heifers, which, in turn, spent more time grazing along the paddock. Dominant animals are known to have priority of access to feed resources (24), and for grazing ruminants, this is related to the priority of access to high-quality grazing areas (9). When the dominant heifers entered the new paddock, they went directly to the feeding area along the fence line and stayed there for a long time, even after all the grain had been consumed. This could represent a strategy to prevent subordinate heifers from eating grain supplement, as they were used to the experimental routine; that is, the grain was offered only once a day.

While the dominant heifers were eating grain supplement, the subordinate animals were grazing. Two key factors that influence the foraging behavior of group-living herbivores are feed availability and individual dominance status; therefore, they weigh the costs and benefits of both when making patch-joining decisions (25). Dominant sheep in heterogeneous flocks use the most preferred areas more intensively, and low-ranked sheep use less preferred areas. However, when high-ranking individuals were removed from the flock, low-ranking sheep shifted their selection patterns by increasing the use of the most preferred areas and strongly avoided using the less preferred sites (26). Manson and Appleby (27) found that cows of similar rank fed together compared with cows of dissimilar rank and that the greatest nearest-neighbor distance was found between animals of low and high rank.

The desire to ingest feed or to avoid disputes with other animals is variable and influences the animal's decision-making. High levels of competition and displacement in the feeder indicate that the access to feed is a priority for cattle (28). Nevertheless, this priority is dependent on its motivation to obtain it (29). Motivation is defined operationally as the tendency for an animal to perform a behavior, but understood as reflecting the animal's desire to do so (30), allowing us to estimate the value that an animal gives to a certain resource after weighing costs and benefits to obtain it. The value an animal gives to a resource is dependent not only on the quality of the resource, but on the need of the animal, as well. In a water restriction situation, subordinate non-lactating cows would drink water every other day, while subordinate lactating cows would fight to drink daily (31).

In this study, when grain supplement was offered at the time of paddock entry (INITIAL), with fresh pasture available, subordinate heifers were motivated to ingest feed, and they could choose to graze along the paddock instead of competing for grain supplement with dominant heifers. However, in the MIDDLE treatment, there were a higher number of agonistic interactions, compared to INITIAL treatment, probably due to the fact that heifers no longer had high-quality pasture available, but still motivated to obtain feed. Therefore, offering the supplement at the time of entering the paddock would reduce fights, giving subordinate heifers an opportunity to graze high-quality pasture, improving their welfare.

Since the subordinate heifers were the first to leave the grain supplement location to graze, they could ingest forage with the same crude protein content as the dominant heifers, while the intermediate heifers were left with pasture of inferior quality. In pasture-based systems, the amount of pasture consumed and its nutritive value may influence the between-cow variability in response to supplement and need to be considered as part of a dynamic model for calculating optimum supplementation rates (32).

In dairy cattle, the first animals moving to an allocation of fresh pasture after a milking session are offered feed of greater nutritive value compared with those arriving last, which is closely related to social hierarchy, as they show a consistent milking order (33). Highly dominant animals may obtain priority in resource access in intensive production conditions (34). Such information can be relatively easy for farmers to collect. Thus, the feeding order can be used as an on-site simple attribute of social dominance in intensive beef cattle production systems (24). Housing and management strategies may be implemented to optimize access to feed and feeding patterns, thus promoting good health, productivity, and welfare (35). Under pastoral systems, synergies between animals' and farmers' grazing decisions have the potential to offer greater benefits to our livestock, our landscape, and ourselves (36).

## Conclusions

Subordinate heifers consistently grazed more and ate less grain supplement than dominant and intermediate heifers. However, when grain supplement was offered at the time animals entered the paddock, more grazing activity took place during supplement feeding, and subordinate heifers could select a high-protein diet. In the INITIAL treatment, this means that subordinate animals could benefit from the better pasture available, keeping a distance from dominant heifers, reducing agonistic interactions, and likely improving their welfare.

## Data Availability Statement

The datasets generated for this study are available on request to the corresponding author.

## Ethics Statement

The animal study was reviewed and approved by Ethics Committee on Animal Use of the Federal University of Santa Catarina (CEUA/UFSC), under the approved protocol number 1004100516.

## Author Contributions

GB and LP contributed to the conception and planning of the experiment. GB, LP, and MH designed the experiment. GB and KS made the data collection and field work. DT did the statistical analysis. GB wrote the manuscript. LP and MH were advisors and revised and helped in writing. All authors have read and approved the last version of the manuscript.

### Conflict of Interest

The authors declare that the research was conducted in the absence of any commercial or financial relationships that could be construed as a potential conflict of interest.
